# Preeclampsia impedes foetal kidney development by delivering placenta-derived exosomes to glomerular endothelial cells

**DOI:** 10.1186/s12964-023-01286-y

**Published:** 2023-11-23

**Authors:** Mengqi Gu, Pengzheng Chen, Dongmei Zeng, Xiaotong Jiang, Qingfeng Lv, Yuchen Li, Fengyuan Zhang, Shuting Wan, Qian Zhou, Yuan Lu, Xietong Wang, Lei Li

**Affiliations:** 1 Department of Obstetrics and Gynaecology, Shandong Provincial Hospital, Shandong University, Jinan, 250021 Shandong China; 2 Department of Obstetrics and Gynaecology, Shandong Provincial Hospital Affiliated to Shandong First Medical University, Jinan, 250021 Shandong China; 3 The Laboratory of Medical Science and Technology Innovation Center (Institute of Translational Medicine), Shandong First Medical University (Shandong Academy of Medical Sciences) of China, Jinan, 250117 Shandong China; 4 Key Laboratory of Birth Regulation and Control Technology of National Health Commission of China, Shandong Provincial Maternal and Child Health Care Hospital, 328 Jingshi East Road, Jinan, 250025 Shandong China

**Keywords:** Preeclampsia, Hypoxia, Reoxygenation, Placenta-derived exosomes, Human glomerular endothelial cell, Foetal renal dysplasia

## Abstract

**Background:**

Foetal renal dysplasia is still the main cause of adult renal disease. Placenta-derived exosomes are an important communication tool, and they may play an important role in placental (both foetal and maternal) function. We hypothesize that in women with preeclampsia, foetal renal dysplasia is impeded by delivering placenta-derived exosomes to glomerular endothelial cells.

**Methods:**

In the present study, we established a PE trophoblast oxidative stress model to isolate exosomes from supernatants by ultracentrifugation (NO-exo and H/R-exo) and collected normal and PE umbilical cord blood plasma to isolate exosomes by ultracentrifugation combined with sucrose density gradient centrifugation (N-exo and PE-exo), then we investigated their effects on foetal kidney development by in vitro, ex vivo and in vivo models.

**Results:**

The PE trophoblast oxidative stress model was established successfully. After that, in in vitro studies, we found that H/R-exo and PE-exo could adversely affect glomerular endothelial cell proliferation, tubular formation, migration, and barrier functions. In ex vivo studies, H/R-exo and PE-exo both inhibited the growth and branch formation of kidney explants, along with the decrease of VE-cadherin and Occludin. In in vivo studies, we also found that H/R-exo and PE-exo could result in renal dysplasia, reduced glomerular number, and reduced barrier function in foetal mice.

**Conclusions:**

In conclusion, we demonstrated that PE placenta-derived exosomes could lead to foetal renal dysplasia by delivering placenta-derived exosomes to foetal glomerular endothelial cells, which provides a novel understanding of the pathogenesis of foetal renal dysplasia.

Video Abstract

**Supplementary Information:**

The online version contains supplementary material available at 10.1186/s12964-023-01286-y.

## AJOG at a Glance

Why was this study conducted?

• Our previous studies have demonstrated that PE has adverse effects on the foetus. However, it is not known how it affects the foetus kidneys. Because placenta-derived exosomes play a key role in maternal-placenta-foetal communication, we aimed to determine the role of placenta-derived exosomes in foetal kidney development in PE patients.

Key Findings

• The total number of exosomes and the number of placenta-derived exosomes in the umbilical cord blood of PE patients were significantly higher than those in normal pregnant women.
- Placenta-derived exosomes from PE patients have adverse effects on foetal kidney development.

What does this add to what is known?

• These findings reveal the critical role of placenta-derived exosomes in fetal kidney growth and development.

## Background

Chronic kidney disease (CKD) is a clinical syndrome characterized by irreversible and progressive impaired renal function [1], affecting approximately 11.7–15.1% of the global population [2]. Due to its high prevalence and the pronounced risks for cardiovascular morbidity and mortality, CKD has become a global public health concern worldwide [3, 4]. Despite extensive efforts, clinical outcomes remain unsatisfactory and the detailed mechanisms of pathogenesis are not yet fully elucidated [5, 6].

In the 1990s, Professor David Barker first systematically expounded the DOHaD theory in The Lancet, describing “the developmental origins of health and disease” [7]. Since then, an increasing number of studies have proven that an adverse intrauterine environment in early life not only affects the growth and development of the foetus but may also cause lasting structural or functional changes, leading to a series of adult diseases in the future [8–10]. Subsequently, Barker et al. showed that adult systolic blood pressure was inversely proportional to birth weight, the intrauterine stage is the critical period for the development of tissues and organs [11]. Human kidney formation begins at 9 and lasts until the end of gestational week 36 [12]. The third trimester of pregnancy is an active period of kidney development, which is susceptible to different harmful factors [13]. Once the normal early-life foetal kidney developments were insulted, it would cause structural and functional changes such as lower nephron numbers in the developing kidney (called renal programming [14]) and progress into renal dysfunction. Therefore, we are aware of the importance of obstetric complications and adverse intrauterine environments in the development of foetal renal dysplasia and decide to further investigate the underlying mechanism to provide more options for diagnosing and treating.

Preeclampsia (PE) is a serious pregnancy complication characterized by the onset of new hypertension after the 20th week of pregnancy [15, 16]. This pregnancy-specific syndrome affects approximately 5–8% of pregnancies worldwide [17]. PE is a lifelong disease for mothers and offspring, with an increased risk of cardiovascular disease, neonatal and child morbidity, mortality and health risks that persist into adulthood [18]. PE plays an important role in intrauterine foetal growth retardation (IUGR) and spontaneous and iatrogenic preterm birth [19]. Although the use of aspirin can partly prevent the occurrence of preeclampsia [20], the most effective cure for preeclampsia is still the delivery of the dysfunctional placenta and baby [21, 22], which will result in preterm birth and low birth weight neonates inevitably. Previous studies have demonstrated that IUGR, preterm birth and low birth weight are key risk factors for chronic kidney diseases [23–25],, while the particular mechanism of foetal renal dysplasia caused by preeclampsia remains unclear.

PE is considered to be a placental disorder, as it can occur in patients with hydatidiform moles [26]. The classical “two-stage” theory reveals that PE is initiated by the placental malperfusion secondary to inadequate trophoblast invasion and insufficient remodeling of uterine spiral arteries, which lead to dysfunction of endothelial cells, followed by the systemic inflammatory response [27, 28]. Unsurprisingly, as the central role in the maternal-foetal interface, the placenta makes adaptative changes, such as inflammation [29], endoplasmic reticulum stress [30] and oxidative stress [31], to participate in the pathogenesis and progression of PE. Meanwhile, placental trophoblasts, representing the major cellular entities of the placenta, also exhibit diminished biological function [32, 33]. Nevertheless, the specific pathways of the dysregulated placenta and trophoblasts leading to foetal renal dysplasia are poorly understood.

Exosomes are 30–100 nm extracellular vesicles that contain a variety of signalling molecules (e.g., proteins, mRNAs, and microRNAs) and are involved in many physiological processes and disease pathogenesis [34, 35]. Maternal-placental-foetal communication is very important for establishing and maintaining a normal pregnancy [36, 37]. Placenta-derived exosomes have been identified in the maternal circulation during pregnancy and participate in the crosstalk between the mother and fetus [38, 39]. As evidenced by previous studies, the concentration of placenta-derived exosomes in maternal circulation increased in early onset-PE but decreased in late onset-PE [40], and lower oxygen tension (1%) can significantly promote the release of exosomes in EVT cultured under 8% oxygen [41]. In maternal circulation, placenta associated exosomal miR-155 was found to inhibit eNOS expression in endothelial cells in PE [42]. In foetal circulation, our previous study first demonstrated that GDM placenta-derived exosomes from umbilical cord plasma could impede foetal lung development [43]. However, little is known about the alteration of placenta-derived exosomes such as concentration, content, and biological functions in PE-associated foetal renal dysplasia.

To our knowledge, placenta-derived exosomes have never been reported in the pathogenesis of PE-associated foetal renal dysplasia. In the present study, we first established a H/R trophoblast model, then isolated and characterized exosomes from the supernatant. By establishing in vitro, ex vivo and in vivo models, we examined the effects of exosomes on foetal kidney development. In parallel, we also show the biological effects of placenta-derived exosomes from PE umbilical cord blood plasma on foetal kidney development. These findings would help further elucidate the pathogenesis of PE-associated foetal renal dysplasia and may provide an effective therapeutic target.

## Methods

### Study population and sample collection protocols

Umbilical cord blood samples (10 from normal pregnancies and 10 from PE) were collected at the Department of Obstetrics of Shandong Provincial Hospital after approval by the Ethics Review Committee of Shandong Provincial Hospital. All patients provided preoperative informed consent. After the myometrium was incised and the placenta was delivered, whole blood was collected by aseptic acupuncture before caesarean section via venepuncture in anticoagulant EDTA-K2 (ethylenediaminetetraacetic acid–K2) and centrifuged at 4000 × g for 15 min. Normal control pregnancies were defined as having no pregnancy complications (*n* = 10, 38.5 ± 0.5 weeks). The PE group was defined as maternal blood pressure (≥ 140/90 mmHg) and urinary protein (≥ 1 +) at 20 weeks of gestation (*n* = 10, 37 ± 1.9 weeks), and those with diabetes and other pregnancy complications were excluded [16, 44]. More clinical information about the sample can be found in Table 1.

**Table 1:** Demographic and clinical characteristics of the study population

Demographic	Normal (*n* = 10)	Preeclampsia (*n* = 10)
Maternal age, y	32.8 ± 3.3	35.8 ± 5.7
Gestational age at delivery, w	38.5 ± 0.5	37 ± 1.9*
Birth weight, g	3355 ± 295.4	2680 ± 813.8****
Systolic blood pressure, mmHg	106.3 ± 8.2	156.1 ± 15.6****
Diastolic blood pressure, mmHg	70.90 ± 8.5	91.9 ± 13.5***
Proteinuria	-	> = + + ****

Data are shown as means ± SDs

All results are reported after adjustment for baseline values using Student’s t tests

*P < 0.05

***P < 0.001

****P < 0.0001

### Cell culture

HTR8/SVneo cells (human first-trimester extravilloustrophoblast cells) were purchased from ATCC. Non-treated (NO) cells were routinely cultured in RPMI 1640 (Gibco) supplemented with 5% exosome-free foetal bovine serum (Gibco) and 1% penicillin/streptomycin at 37 °C and 5% CO_2_. Hypoxia/reoxygenation (H/R) was performed as previously described [45]. The trophoblast cells were cultured in two cycles: the first was in a hypoxic environment in a tri-gas cell culture incubator flushed with 2% O_2_ for 8 h, and this was followed by reoxygenation in a standard incubator with 20% O_2_ for 16 h.

Human glomerular endothelial cells (HGECs) were purchased from ATCC. The cells were routinely cultured in DMEM (Gibco) with 10% exosome-free FBS and 1% penicillin/streptomycin at 37 °C and 5% CO_2_.

### ELISA

ELISA for human HIF-1α were performed according to the manufacturer’s instructions. In brief, HTR8/SVneo cell lysates buffer (NO and H/R) were collected, and the precipitate was removed by centrifugation for 10 min at 4 °C incubated in a 96-well plate precoated with capture antibodies. Samples were added to the plate. Wells were washed 5 times and incubated with a secondary antibody conjugated to horseradish peroxidase. Then the substrate solution was added, and the optical density was determined at 450 nm. The protein levels were calculated using a standard curve derived from known concentrations of the respective recombinant proteins.

### Exosomes isolation

Trophoblast-derived exosomes from HTR8/SVneo cells were extracted by ultracentrifugation [46, 47]. In simple terms, the culture medium was centrifuged at 4 °C at 500 × g, 2000 × g and 12,000 × g for 10 min, 30 min and 45 min, respectively, to remove whole cells and debris. The resulting supernatant was sterilized by means of a 0.22-μm filter and centrifuged at 120,000 × g (Hitachi CP100MX) for 70 min. The particles were resuspended in PBS and centrifuged after washing (120 000 × g, 70 min). Finally, the exosomes were suspended in 200 µl of PBS and placed in a -80 °C freezer for subsequent experiments. The concentration of exosomes was measured with a BCA protein analysis kit (Solarbio, Beijing, China).

Placenta-derived exosomes from cord blood were extracted by a series of sucrose density gradient centrifugations and ultracentrifugation [42, 43]. In simple terms, 20 ml of cord blood was centrifuged at 4 °C at 3000 × g for 20 min to produce 8 ml plasma. Next, an equal volume of 1 × PBS was added and centrifuged at 4 °C at 500 × g, 2000 × g and 12,000 × g for 15 min, 30 min and 45 min, respectively. The supernatant was taken, and the precipitate was discarded to remove cells, cell fragments, large vesicles and small vesicles. The supernatant was filtered through a 0.22-µm membrane, and the supernatant mixture was suspended in 0.25 M-sucrose. The suspension was then stratified on a sucrose density gradient, centrifuged at 4 °C at 200 000 × g for 16 h, and divided into six fractions: F1, 1.03; F2, 1.06; F3, 1.09; F4, 1.11; F5, 1.14; and F6, 1.18 g/ml. The fractions were centrifuged at 4 °C and 120,000 × g for 2 h. Then, in order to enrich placenta-derived exosomes in umbilical cord blood, we collected F2 and F3 exosomes and suspended them in 200 µl PBS for subsequent experiments. The concentration of exosomes was measured with a BCA protein analysis kit (Solarbio, Beijing, China).

### Transmission electron microscopy

Exosomes (10 μl) were added to copper wire for precipitation for 1 min, and the floating liquid was absorbed on filter paper. Ten microlitres of phosphotungstic acid was added to the copper wire for precipitation for 1 min, and the floating liquid was absorbed by filter paper. After drying for several minutes at room temperature, TEM imaging results were obtained at 100 kV (Xiuyue Biol, Jinan, China).

### Nanoparticle tracking analysis

Ten microlitres of exosome sample was removed and diluted to 30 μl. The instrument performance test was first performed with the standard product. After passing the test, the exosome sample was loaded. The particle size and concentration of exosomes detected by the instrument were obtained after the samples were tested (Xiuyue Biol, Jinan, China).

### Exosome labelling

The exosomes were labelled with the fluorescent dye PKH67 (green) (PKH67; Sigma) and incubated for 20 min according to the manufacturer’s protocol. The 17-labelled excised suspensions were filtered with a 100-kDa intercepted hollow fibre membrane to remove excess dye. HGECs were inoculated in 96-well plates and incubated with double-labelled exosomes (100 μg/ml) for 24 h. Kidney explants were inoculated in 24-well plates and incubated with double-labelled exosomes (200 μg/ml) for 96 h. HGECs were fixed in 4% paraformaldehyde for 10 min, 100 μl of diluted phalloidin was added to each well and incubated at room temperature for 30 min, and DAPI staining solution was added for 5 min. Then, labelled cells were prepared and observed under ImageXpress Microconfocal with MetaXpress software (overall magnification 100X).

To fluorescently label exosomes for in vivo imaging, we resuspended the pellet centrifuged at 100,000 × g for 2 h in 7.0 ml of 7.5 µM DiR (Life Technologies, Carlsbad, CA, USA) in PBS. After mixing, the exosomes were incubated in the DiR/PBS solution for 15 min at room temperature in the dark and then ultracentrifuged at 100,000 × g for 1 h. The final pellet was resuspended in 50 µl of PBS and stored at − 80 °C.

### EdU staining

EdU staining was performed according to the manufacturer's instructions (Beyotime, Shanghai, China). Briefly, HGECs (5 × 10^3^ cells/well) were incubated with different exosomes (NO-exo, H/R-exo, N-exo, PE-exo, 100 μg/ml) for 24 h. EdU (10 μM/well) was then added to the medium and incubated for 4 h. After labelling, the cells were washed three times with PBS and then fixed with 4% formaldehyde. After incubation with glycine, the cells were washed with PBS containing 0.5% Triton X-100. After the nuclei were stained with DAPI, cell proliferation was observed by ImageXpress Micro Confocal with MetaXpress software (overall magnification 200X).

### Tube formation assay

To quantitatively determine the ability of HGECs to generate blood vessels in vitro, we applied substrate glue to the bottom of 96-well plates. HGECs (1 × 10^4^ cells/well) were added to serum-free endothelial cell culture medium after Matrigel mix (BD Bioscience) coagulation, and the cells were incubated with different exosomes (NO-exo, H/R-exo, N-exo, PE-exo, 100 μg/ml) for 6 h. Finally, the cells were photographed with an inverted microscope (Thermo) for analysis; ImageJ was used to quantify tube formation.

### Cell migration assay

The migration of HGECs was measured using Transwell inserts (Corning, USA) with 8 μm polycarbonate membranes. The process was as follows: 200 µl of serum-free medium was added to the upper chamber, and 650 µl of complete medium containing 5% serum was added to the lower chamber. HGECs (1 × 10^4^ cells/well) were incubated in the upper chamber with exosomes from the different groups (NO-exo, H/R-exo, N-exo, PE-exo, 100 μg/ml). After 24 h of culture, the cells that migrated to the lower chamber were stained with crystal violet. Finally, an inverted microscope (Olympus, Tokyo, Japan) was used at a magnification of 200X to count the average number of migrated cells.

### Cell permeability assay

The flow of Evans blue bound to albumin across the monolayer of a functional artificial liver was measured by spectrophotometry using a modified two-compartment model that was described previously for quantitative permeability [48]. In brief, HGECs were plated (5 × 10^4^ cells/well) in a Transwell tube with diameters of 0.4 μm and 6.5 mm for 3 days. Confluent monolayers were incubated with different exosomes (NO-exo, H/R-exo, N-exo, PE-exo, 100 μg/ml) for 24 h. The inserts were washed with PBS (pH 7.4), and then, 0.5 ml (0.67 mg/ml) of Evans blue BSA (4%) diluent was added to the medium. Fresh medium was added to the lower chamber, and Evans blue BSA was added to the upper chamber. After 10 min, the optical density at 650 nm in the lower chamber was measured. The experiment was repeated several times in triplicate.

### Animal study

C57 male and female mice (6–8 weeks) were purchased from Jinan Pengyue Experimental Animal Breeding Company and kept in a temperature-controlled room at 24 °C with a light/dark cycle of 12:12 h and free access to food and water. This project was implemented in accordance with the Animal Protocol procedures approved by the Department of Laboratory Animal Science, Shandong University Affiliated Provincial Hospital Animal Laboratory, and animals were processed in accordance with guidelines published in the National Institutes of Health under the guidance of the Animal and Institutional Animal Health and Use Committee. To evaluate the gestational age of mouse embryos consistently and accurately, we paired male and female C57 mice for one night. The first vaginal plug was found on embryonic day 0.5 (E0.5).

### Foetal kidney culture in vitro

Foetal kidneys of E12.5 mice were isolated in vitro and randomly divided into different group as described previously [49–51]. In brief, the foetal kidney was transferred to a Transwell semipermeable membrane, and 500 µl of DMEM/F12 culture medium was added to each well and incubated in an incubator. On the second day, the old culture medium was discarded, and NO-exo, H/R-exo, N-exo and PE-exo were added (200 µg/ml, 3 multiple wells/group). The kidney was cultured in the incubator for another 4 days, and the growth of the kidney was observed by taking pictures with an inverted microscope. The ureteral buds (E-cadherin, green) and glomerulus (WT-1, red) of foetal kidneys cultured for 5 days were stained by immunofluorescence and photographed by a laser confocal microscope to observe the structural changes of foetal kidney development. Foetal kidney protein and RNA were extracted, and the growth and development of the foetal kidney were quantitatively analysed by Western blot analysis and qPCR.

### Amniotic cavity injection

For confirmation that exosomes could enter the foetus, E14.5 mice were continuously anaesthetized with isoflurane, and the mice received minor open surgery and intrauterine injection of DIR-labelled exosomes as described previously [52]. Twenty-four hours later, the animal’s lateral abdomen was captured by an in vivo imaging system (Tanon ABL X6, China). After in vivo imaging, the animals were sacrificed by inhaling carbon dioxide in accordance with IACUC and American Veterinary Medical Association guidelines.

Pregnant mice with similar body weights on the same day were randomly divided into different group. NO-exo, HR-exo, N-exo and PE-exo (500 µg/ml) were injected into the amniotic cavity of pregnant mice on E14.5. The foetal mice were removed by caesarean section on E18.5, and the kidneys were separated. PAS staining and immunohistochemistry staining were performed to observe the structural changes during foetal kidney development. Foetal kidney protein and RNA were extracted, and the growth and development of the foetal kidney were quantitatively analysed by Western blot analysis, IHC and qRT-PCR.

### Western blot analysis

Total protein was extracted from HTR8/SVneo cells, exosomes or HGECs, explants, and kidneys treated with exosomes. The total protein concentration was measured with a BCA kit. Next, the proteins were separated by gel electrophoresis and then transferred to polyvinylidene fluoride membranes using electrical blotting. Ten micrograms of total protein from each sample was used for HIF1-α, Golgi marker, exosome-specific antibodies and junction protein-specific antibodies. The antibody HIF1-α (1:1000; Abcam). The Golgi marker GM130(1:1000; Abcam). The exosome-specific antibodies included CD63 (1:1000; Abcam) and TSG101 (1:1000; Abcam); the placenta-specific antibody was PLAP (1:1000; Abcam). The junction protein-specific antibodies included VE-cadherin (1:1000; CST) and Occludin (1:1000; Abcam) and the housekeeping antibodies included β-actin (1:1000; CST). The antibodies were incubated with the blots overnight at 4 °C. The blots were then incubated with HRP-conjugated goat anti-rabbit secondary antibodies (Proteintech, Rosemont, IL) at room temperature for 1 h. The immunofluorescence bands were detected with a kit (Merck Millipore, Burlington, MA), and the intensity of the bands was quantified by an Amersham Imager 600 Imaging System (GE Healthcare, Chicago, IL).

### Quantitative RT-PCR (qRT-PCR) analysis

HGECs, explants, and kidneys were treated with different exosomes (NO-exo, H/R-exo, N-exo, PE-exo). A StepOnePlus real-time quantitative PCR system (Invitrogen, CA, USA) was used to measure mRNA. The VE-cadherin primers (5´-ACGACAACTGGCCTGTGTTCAC and 3´-TG-CATCCACTGCTGTCACAGAG) yielded a 101-base pair fragment. The Occludin primers (5´-ACCCCCATCTGACTATGTGGAA and 3´-AGGAACCGGCGTGGATTTA) yielded a 115-base pair fragment. The Gapdh primers (5´-AGATCCCTCCAAAATCAAGTGG and 3´-GGCAGAGATGATGACCCTTTT) yielded a 130-base pair fragment. SYBR® Premix Ex Taq™ (Accurate Biotechnology, Hunan, China) was used for amplification, and gene expression was calculated with the 2 − ΔΔCT method (with Gapdh as an internal reference).

### Immunofluorescence, periodic acid-schiff, and immunohistochemistry

Immunofluorescence was used to detect the expression of ureteral buds and glomeruli in explants. The tissues were fixed with 4% paraformaldehyde for 48 h and treated as usual. For paraffin-embedded tissue, sections of 3–5 μm thick paraffin-embedded tissue were cut and dewaxed with xylene, dehydrated with ethanol, and stained with periodic acid-schiff (PAS). The expression of VE-cadherin and Occludin in tissue sections was detected by immunohistochemistry (IHC).

### Statistical tests

The data are expressed as the means ± SDs of three independent experiments. ImageJ software was used for data analysis, and GraphPad Prism (ver. 7; GraphPad Software, Inc., La Jolla, CA) was used for statistical analysis. Student’s t tests were used to analyse differences between the two groups. *P* < 0.05 was considered statistically significant (ns, *P* > 0.05, *P < 0.05, **P < 0.01, ***P < 0.001, ****P < 0.0001).

## Results

### HIF1-α expression, exosomes identification

**Fig. 1:**
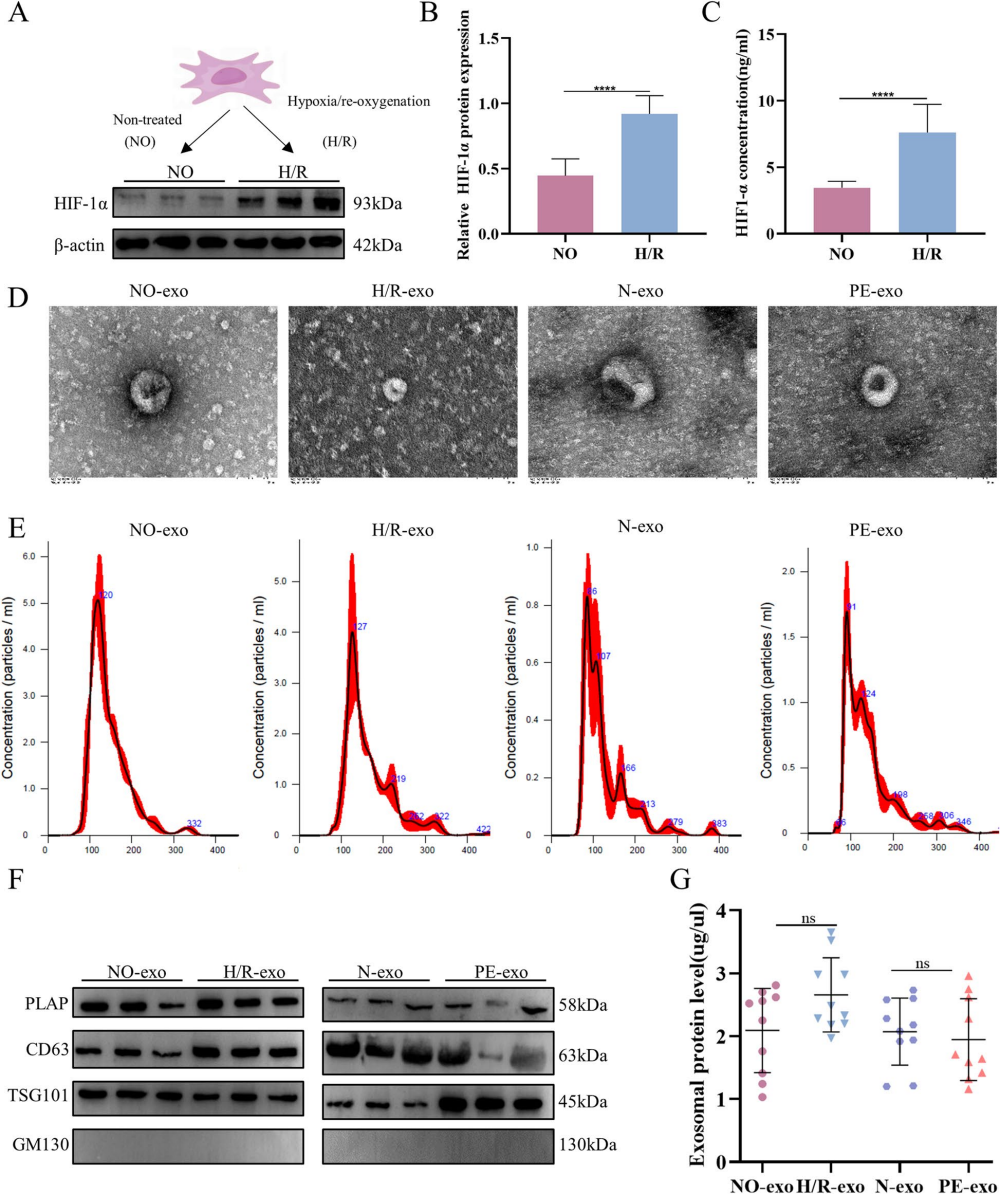
HIF1-α expression and exosomes identification. **A** and **B** Western blot of HIF1-α protein expression. **C** ELISA of HIF1-α concentration. **D** TEM image of exosomes, Scale: 100 nm. **E **NTA results showing the diameters of exosomes. **F** Western blot showing positive results for the specific placenta-derived exosome markers CD63, TSG101, and PLAP, and negative results of Golgi marker GM130. **G** Exosomes concentrations of NO-exo, H/R-exo, N-exo and PE-exo. Values are presented as means ± SD, ns, *P* > 0.05, ***P < 0.001, ****P < 0.0001

HIF1-α expression is increased under H/R conditions [53, 54]. To clarify whether the oxidative stress model was successfully constructed, we analysed HIF1-α expression in HTR8/SVneo cells under NO and H/R conditions. As expected, WB (Fig. 1A and B) and ELISA (Fig. 1C) results showed increased HIF1-α expression following H/R conditions.

Compared with normal pregnancies (*n* = 10), women with PE(*n* = 10) had higher blood pressure and proteinuria, lower foetal weight, and earlier weeks of termination, as shown in the table. Transmission electron microscopy (TEM) revealed heterogeneous populations of exosomes with typical cup shapes ranging from approximately 30–100 nm in diameter (Fig. 1D). Exosomes were characterized according to their size and concentration by nanoparticle size analysis (NTA), and the concentrations and particle sizes of different samples were uniform, with an average particle size of 146.6 ± 3.9 nm (Fig. 1E). Western blot analysis confirmed the expression of exosomes (CD63, TSG101, PLAP), and negative expression of the Golgi marker GM130 (Fig. 1F). In addition, we found that the different exosomes protein concentrations have no significant difference (Fig. 1G).

### Function of trophoblast-derived exosomes on HGECs

**Fig. 2:**
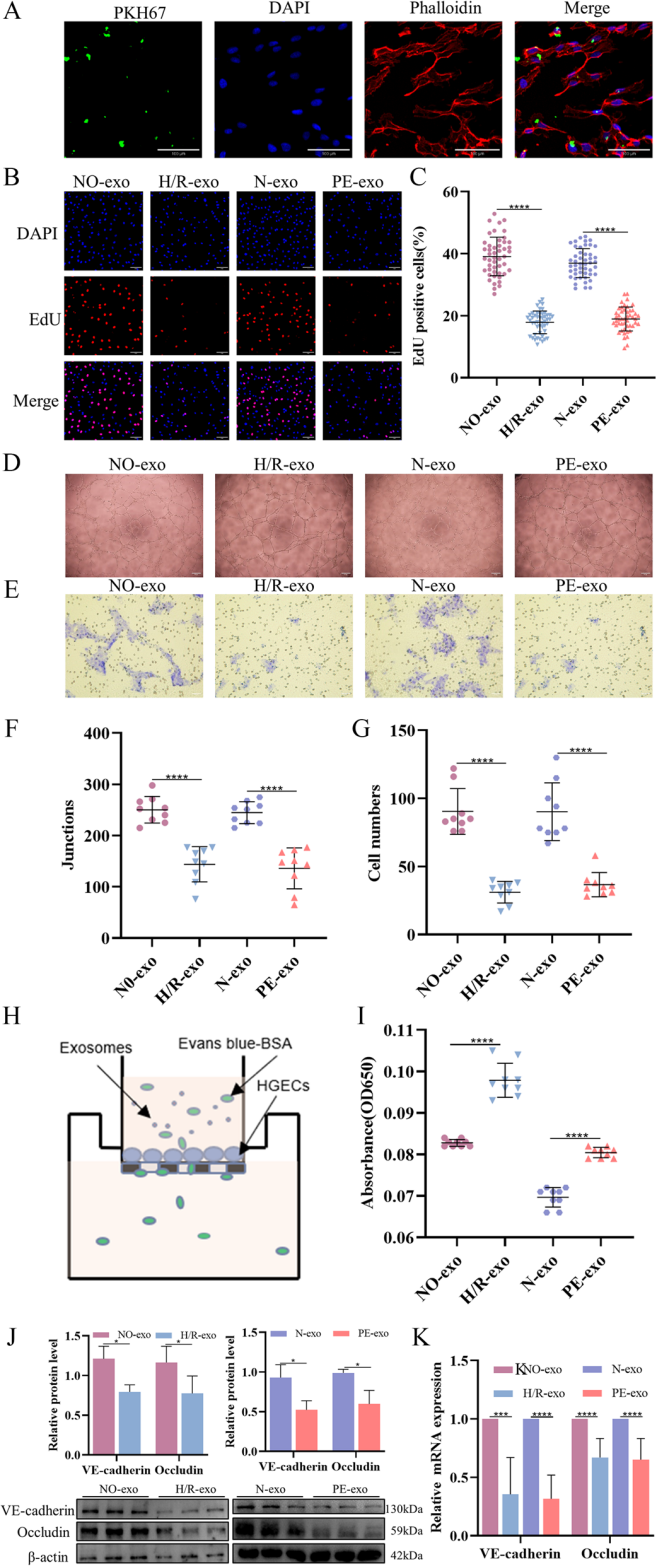
Function of trophoblast-derived exosomes on HGECs. **A** Confocal microscopy showed that PKH67-labelled exosomes were internalized by HGECs, Scale: 100 μm. **B** and **C** EdU staining was used to examine proliferation after exosomes (100 μg/ml) treatment, Scale: 50 μm. **D** and **F** The reaction of cultured HGECs to exosomes (100 μg/mL) was examined in the angiogenesis assay, Scale: 200 μm. **E** and **G** Transwell experiments were used to examine HGECs migration after incubation with exosomes (100 μg/mL), Scale: 200 μm. **H** and **I** The endothelial monolayer barrier function of cells was tested in the model after treatment with exosomes (100 μg/ml). **J** Western blot analysis of the expression of VE-cadherin and Occludin in exosomes. **K** qRT-PCR of the expression of VE-cadherin and Occludin in exosomes. Values are presented as means ± SD, ***P < 0.001, ****P < 0.0001

Preeclampsia can damage endothelial cells and lead to multiple organ dysfunction. Therefore, HGECs were selected to study the effects of PE- related exosomes on the kidney. The pKH67-labelled exosomes were incubated with HGECs for 24 h, and most of the recipient cells showed PKH67 fluorescence (Fig. 2A). A series of cell function experiments on HGECs treated with exosomes. Figure 2B, C shows that both H/R-exo and PE-exo can inhibit HGECs proliferation compared with NO-exo and N-exo. H/R-exo and PE-exo significantly inhibited tube formation and cell migration (Fig. 2D, E, F, and G). In addition, H/R-exo and PE-exo significantly reduced the barrier function of endothelial cells, leading protein leakage increased (Fig. 2H, I). WB and qRT-PCR results showed that the levels of VE-cadherin and Occludin in the H/R-exo and PE-exo groups were significantly reduced compared with those in the NO-exo and N-exo groups (Fig. 2J, K). These data suggest that exosomes can be internalized by HGECs, and that PE-related exosomes can inhibit cell proliferation, tube formation, and migration, reduce cell barrier function and decrease cell junction protein expression.

### Function of trophoblast-derived exosomes on kidney explants

**Fig. 3:**
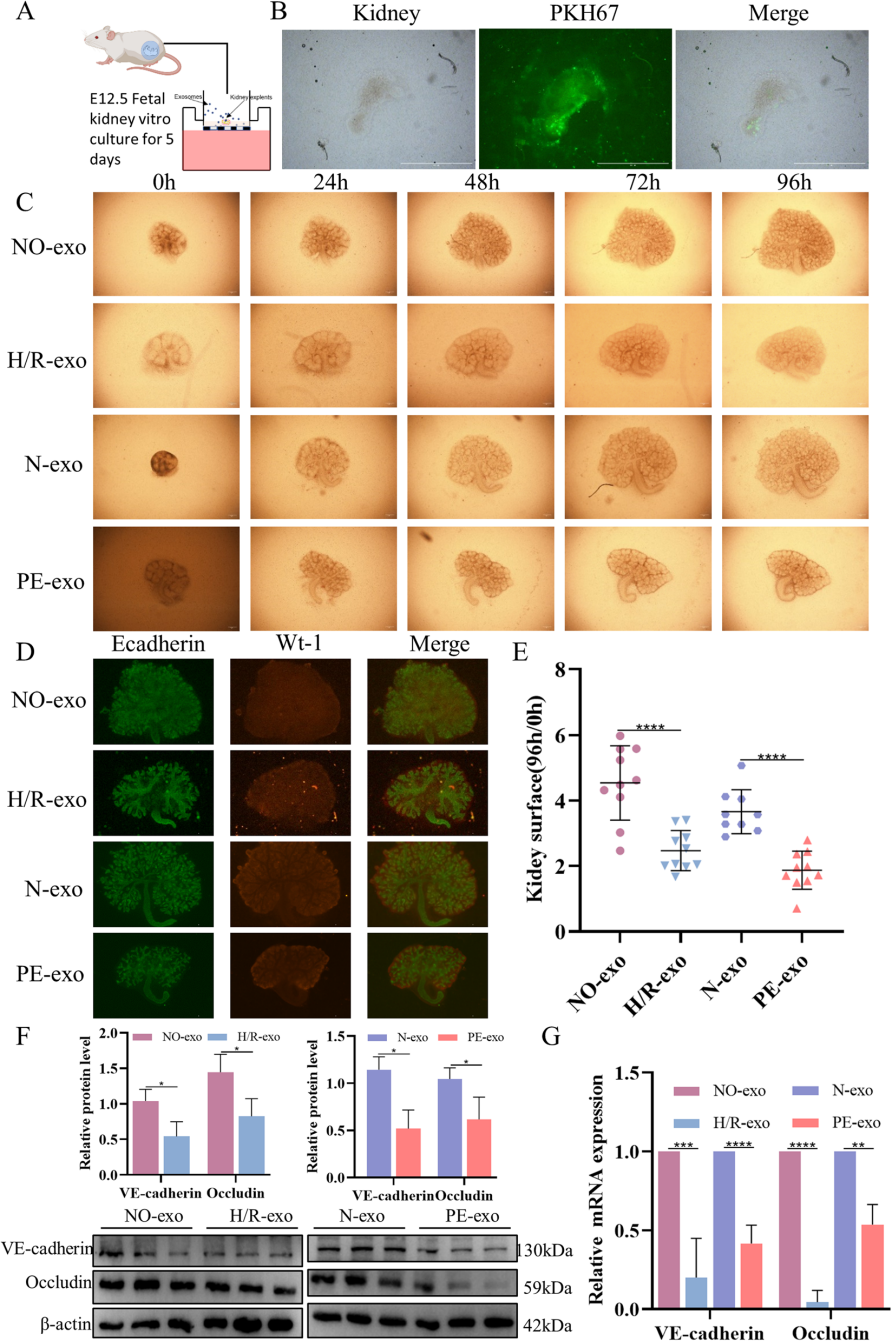
Function of trophoblast-derived exosomes on kidney explants. **A** Schematic diagram of the culture process. **B** Fluorescence microscopy showed that PKH67-labelled exosomes were internalized by kidney explants, Scale: 500 μm. **C** Microscopy showed kidney explants growth situation, Scale: 200 μm. **D** Fluorescence microscopy showed kidney explants, Scale: 500 μm. **E** Kidney explants growth surfaces. **F** Western blot analysis of the expression of VE-cadherin and Occludin; G, qRT-PCR of the expression of VE-cadherin and Occludin. Values are presented as the means ± SD, **P < 0.01, ***P < 0.001 ****P < 0.0001

Given the adverse effects of H/R-exo and PE-exo on HGECs, we explored the effects of H/R-exo and PE-exo on kidney development. An in vitro exosome exposure model of foetal kidney explants was used to observe the morphological changes of the kidney explants. In Fig. 3A, exosomes were added to the foetal kidneys of E12.5 isolated ex vivo. The pKH67-labelled exosomes were incubated with explants for 24 h, and most exosomes were observed in explants (Fig. 3B). After 96 h of culture, the growth and branch formation of kidney explants treated with H/R-exo and PE-exo were inhibited compared to the NO-exo and N-exo groups (Fig. 3C, D and E). WB and qRT-PCR results showed that the levels of VE-cadherin and Occludin in the H/R-exo and PE-exo groups were significantly reduced compared with those in the NO-exo and N-exo groups (Fig. 3F and G). These data suggest that PE-related exosomes are detrimental to the growth, branching morphogenesis and maturation of renal explants.

### Function of trophoblast-derived exosomes on foetal mouse kidney

**Fig. 4:**
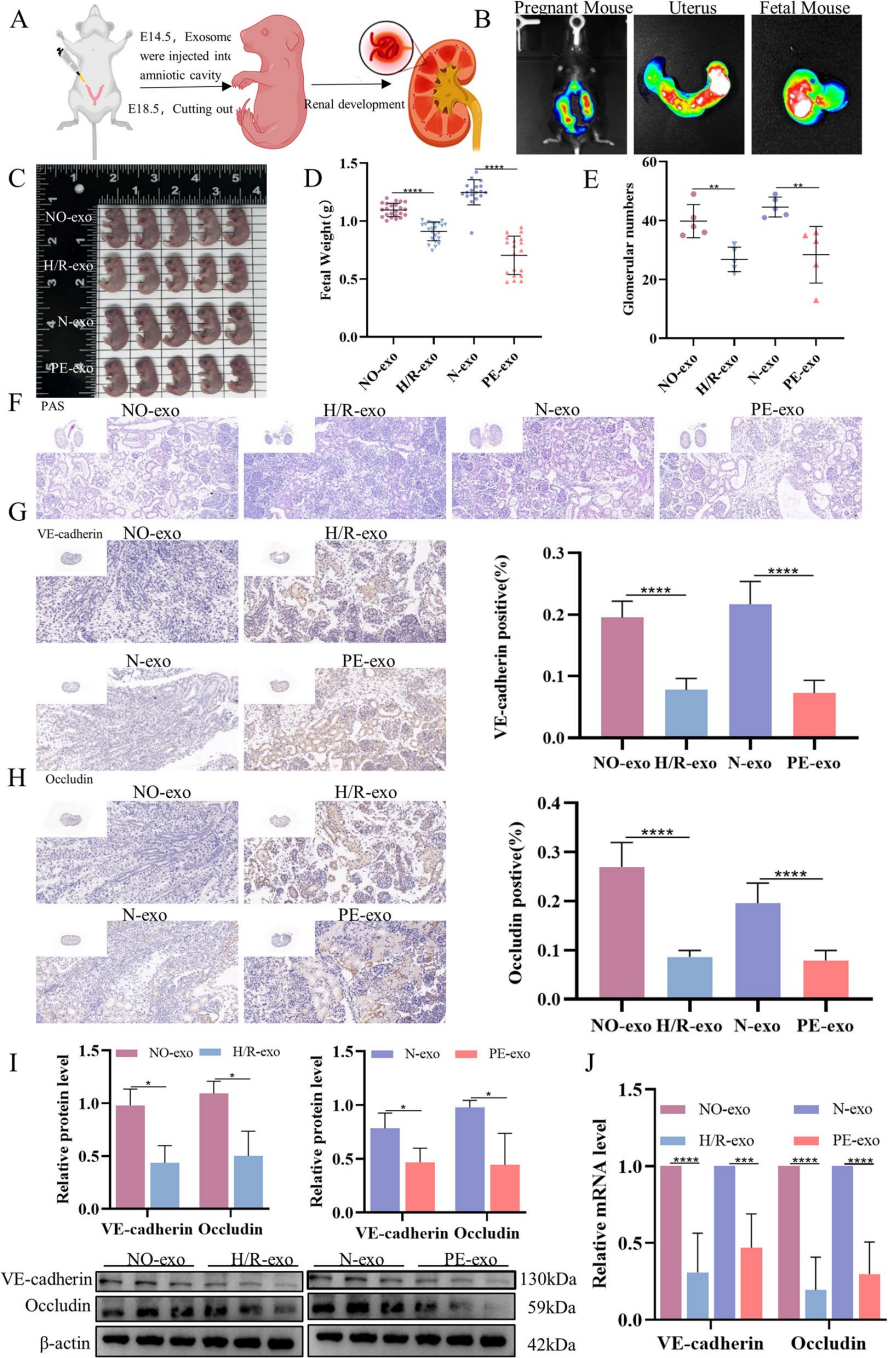
Function of trophoblast-derived exosomes on foetal mouse kidney. **A** Schematic diagram of culture process. **B** In vivo imaging system showed that DIR-labelled exosomes were internalized by Fetal mouse. **C** Appearance of fetal mice. **D** Foetal mice weight. **E** Glomerular numbers of foetal mice. **F** Staining of kidney segmens with PAS, Scale: 50 μm. **G** IHC of the expression of VE-cadherin, Scale: 20 μm. **H** IHC of the expression of Occludin, Scale: 20 μm. **I** Western blot of VE-cadherin and Occludin. J, qRT-PCR of the expression of VE-cadherin and Occludin. Values are presented as means ± SD, **P < 0.01, ***P < 0.001 ****P < 0.0001

To further investigate the effect of exosomes on foetal kidney development in vivo, different exosomes were injected through the amniotic cavity into pregnant C57BL/6 J mice at E14.5 (Fig. 4A). As shown in Fig. 4B, DiR-labelled exosomes were obviously detected in foetal mice. We found that foetal body weight (Fig. 4C and D), and glomerular number (Fig. 4E and F) were significantly lower in the H/R-exo and PE-exo groups than in the NO-exo and N-exo groups. IHC, WB, and qRT-PCR results showed that the levels of VE-cadherin and Occludin in the H/R-exo and PE-exo groups were significantly lower than those in the NO-exo N-exo groups (Fig. 4G, H, I and J). These data suggest that PE-related exosomes can delay foetal kidney development and maturation in vivo.

## Discussion

In the 1990s, Professor David Barker wrote an article in The Lancet, the first systematic elaboration of DOHaD theory [8]. Since then, an increasing number of studies have provn that an adverse intrauterine environment in early life is not only unfavour to the growth and development of the foetus, but may also cause continuous changes in the structure or function of the foetus, which may lead to a series of adult diseases [23, 55].

The intrauterine stage is a critical period for kidney development. At the 9th week of gestation, kidney formation begins, and this developmental process continues until the 36th week of gestation [12]. The active period of kidney development is the third trimester of pregnancy, during which various harmful factors can affect kidney development [56, 57]. Poor development of the kidney, abnormal activity of the sympathetic nerve and disturbance of endothelial function can increase susceptibility to hypertension in adulthood [57]. In conclusion, poor kidney development during pregnancy, especially at the end of pregnancy, may be the beginning of adult chronic kidney disease in the offspring of PE patients.

PE is a serious complication of pregnancy characterized by new-onset hypertension after the 20th week of gestation. This pregnancy-specific syndrome affects approximately 5–8% of pregnancies worldwide [58]. It can have long-term effects on the mother and offspring, with increased susceptibility to cardiovascular diseases, neonatal and childhood morbidity, mortality, and long-term effects that can persist into adulthood [59]. Reportedly, oxidative stress is a key link in the pathogenesis of preeclampsia [60]. Long-term placental ischaemia and hypoxia can lead to the dysfunction of the trophoblast invasion phenotype, which leads to the blockade of spiral artery remodelling and a decrease in placental perfusion, and inducing preeclampsia [61]. Trophoblasts caused by oxidative stress can mimic the preeclamptic “impaired function”. Therefore, trophoblasts caused by oxidative stress have been used to study the pathogenesis of PE [62, 63].

Exosomes are 30–100 nm extracellular vesicles that contain a variety of signalling molecules, such as proteins, mRNA, and miRNA, and are involved in many physiological processes and disease pathogenesis [64]. Maternal-placenta-foetal communication is important for establishing and maintaining a normal pregnancy [36]. Previous studies have shown that placental-derived exosomes play an important role in maternal-placenta-foetal communication. There are placenta-derived exosomes in maternal peripheral blood, and there are significant differences between pregnant women and nonpregnant women. In addition, placenta-derived exosomes also play an important role in pathological pregnancy [34].

PE can lead to foetal renal dysplasia, which is the main cause of adult kidney disease, but the specific mechanism is not clear [65, 66]. Therefore, based on previous studies, we comprehensively used molecular biology, cell biology, explant culture, animal experiments and other methods to investigate the scientific hypothesis that placenta-derived exosomes, as a bridge of cell communication, play an important role in PE-induced foetal renal dysplasia.

Reportedly, placenta hypoxia and oxidative stress are key markers in the pathogenesis of preeclampsia, which can lead to the dysfunction of the trophoblast invasion phenotype, inadequate spiral artery remodeling and decreases in placental perfusion [60, 61, 67]. Previous studies have demonstrated that subjecting the trophoblast under H/R conditions could mimic PE in vitro [62, 63]. Upon hypoxia conditions, the HIF-1α subunit becomes stable and can interact with coactivators to promote its activity [68]. Also, a previous study demonstrated that the expression of HIF-1α was upregulated in PE patients and hypoxia-exposed trophoblasts. Meanwhile, elevated HIF-1α can lead to trophoblast dysfunction and PE. In our study, we cultured the HTR8/SVneo cells under hypoxia and reoxygenation conditions. The WB and ELISA results showed the upregulation of HIF-1α in protein and supernatant, which indicated that we established an in vitro model of PE successfully.

Subsequently, we collected the HTR8/SVneo supernatant to isolate exosomes by ultracentrifugation. Additionally, we isolated placenta-derived exosomes from umbilical cord blood plasma of PE patients to explore the effects of placenta on foetal kidney development comprehensively. However, placenta-derived exosomes are a heterogeneous group of exosomes secreted by various placental cells, and most of them are released by the syncytiotrophoblastic layer [69]. Thus, we isolated placenta-derived exosomes by ultracentrifugation combined with sucrose density gradient centrifugations to achieve enrichment and purification. Then, the trophoblast-derived and placenta-derived exosomes were identified by NTA, TEM and WB. The results showed that the diameter of placenta-derived exosomes was 30–100 nm, with a typical cup-shaped bilayer membrane structure. The expression of PLAP, TSG101 and CD63 was positive, and the expression of GM130 was negative. Previous studies found that the concentration of placenta-derived exosomes in maternal circulation increased in early onset-PE but decreased on late onset-PE [40], and lower oxygen tension (1%) can significantly promote the release of exosomes in EVT cultured under 8% oxygen [41]. However, several studies demonstrated there are no significant differences between normal and PE pregnancies [37, 70]. Our study find that the concentration of different exosomes has no significant difference.

Chronic kidney disease is characterized by reduced glomerular filtration rates and increased urinary albumin excretions [71]. Barrier dysfunction of HGECs, leading to protein leakage and increased permeability, is a key factor in kidney development and chronic kidney disease [72]. To investigate the effects of placenta-derived exosomes on kidney development, we performed a series of in vitro cell experiments on HGECs. Immunofluorescence showed that exosomes could enter HGECs. At the same time, compared to the NO-exo and N-exo groups respectively, the H/R-exo and PE-exo groups exhibited dysfunction of glomerular endothelial cells by inhibiting their proliferation, migration, tube formation, and monolayer filtration membrane barrier ability. In addition, the qRT-PCR and WB results showed that the mRNA and protein levels of VE-cadherin and Occludin in the H/R-exo and PE-exo groups were lower than those in NO-exo and N-exo groups, suggesting that the barrier function of HGECs were impaired.

In recent years, the application of embryonic kidney ex vivo culture technology in kidney development has received extensive attention [73]. To further investigate the role of placenta-derived exosomes in foetal kidney development, foetal mouse kidney explants were cultured ex vivo for 5 days. The growth and development of the foetal mice kidney explants were observed under a microscope. Immunofluorescence results showed that exosomes could enter the fetal mouse kidney explants. Compared with those in the NO-exo and N-exo groups, the kidney explants in the H/R-exo and PE-exo groups developed poorly and the growth area was restricted. In addition, the qRT-PCR and WB results showed that VE-cadherin and Occludin in the H/R-exo and PE-exo groups were lower than those in the NO-exo and N-exo groups at both the transcriptional and protein levels, which was consistent with the HGECs experiment, indicating that the barrier function of the foetal mice kidney explants was impaired.

Increasingly, plenty of studies have found that PE patients have poor maternal and foetal outcomes, and their preterm birth rates and stillbirth rate are higher. Most foetuses have low body weight, poor growth, and damage to the offspring’s cardiovascular system and urinary system [74–76]. The role of placenta-derived exosomes in foetal kidney development was further investigated by intra-amniotic injection of placenta-derived exosomes in pregnant mice. Small animal in vivo imaging can show that placenta-derived exosomes can enter the foetal mice. Through morphological observation, we found that fetal mice in the H/R-exo and PE-exo groups grew and developed worse than those in the NO-exo and N-exo groups, specifically as shown by reduced foetal weight, and reduced kidney weight. Thirty-five years ago, Brenner found that the number of nephrons was associated with renal function [77]. Fewer nephrons represent a diminished filtration surface area, leading to a reduction of sodium excretion and renal adaptive capacity [23]. Our PAS staining results showed that the number of glomeruli in the H/R-exo and PE-exo groups was lower than that in NO-exo and N-exo groups. In addition, the qRT-PCR and WB results showed that the mRNA and protein levels of VE-cadherin and Occludin in the PE group were lower than those in the normal group, which was consistent with the experimental results of HGECs and foetal rat kidney explants, further indicating that the barrier function of the foetal kidney is impaired.

**Fig. 5:**
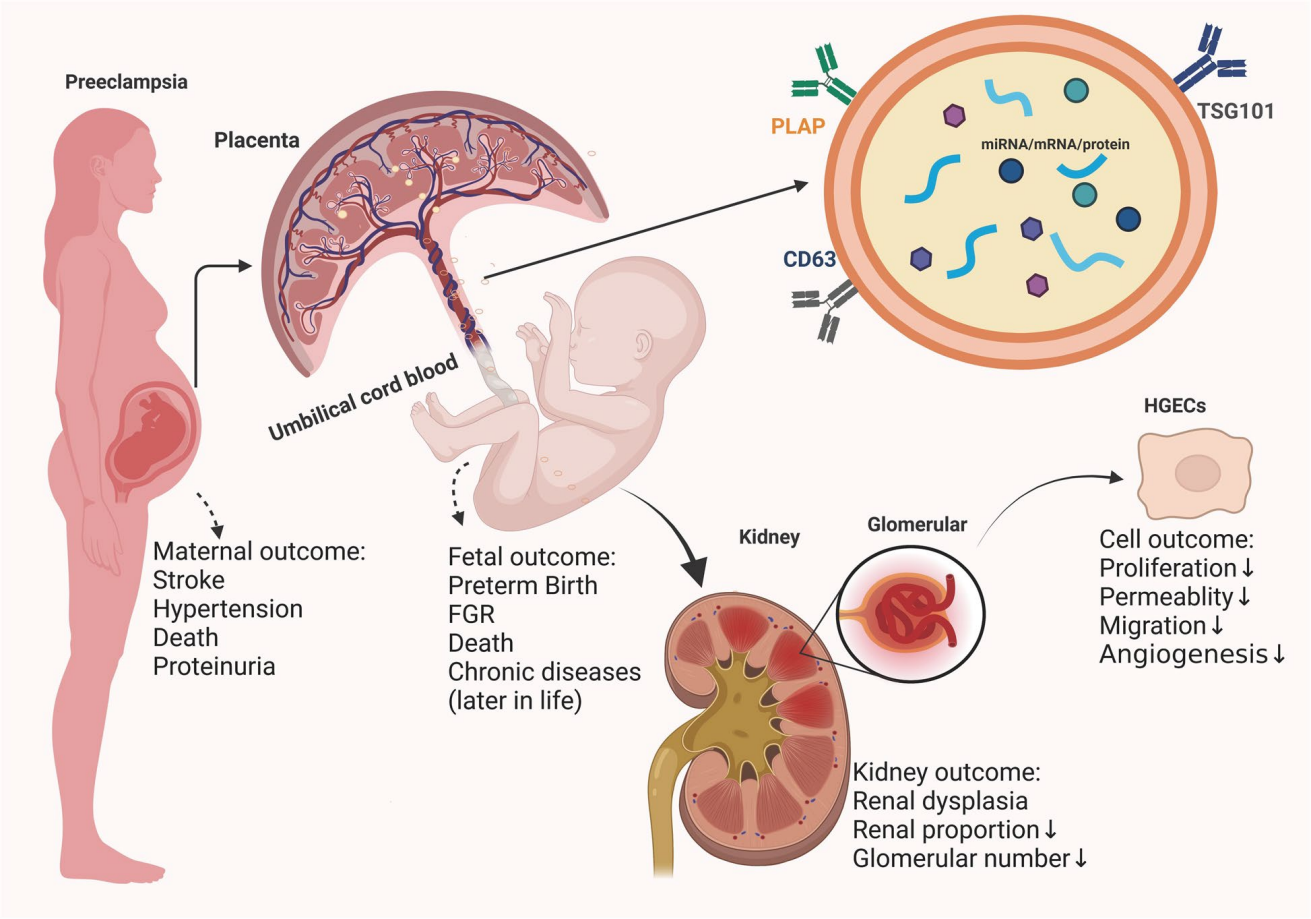
Schematic diagram of the experimental process

In summary, we suggest that placenta-derived exosomes, as a bridge of cell communication, play an important role in PE-induced foetal renal dysplasia, and the underlying mechanism still needs to be further studied. Inhibitors of related pathways mediated by placental exosomes may be potential targets for the treatment of foetal kidney dysplasia caused by PE (Fig. 5).

## Conclusions

In the present study, we found that NO-exo, N-exo, H/R-exo and PE-exo can enter glomerular endothelial cells and that H/R-exo and PE-exo damage glomerular endothelial cell function compared with NO-exo and N-exo. In addition, our study showed that H/R-exo and PE-exo could lead to foetal dysplasia and foetal kidney dysplasia in comparison with NO-exo and N-exo.

## Data Availability

The datasets generated and analysed during the current study are are available from the corresponding author on reasonable request. All data included in this study are available upon request by contact with the corresponding author.

## Abbreviations

PE: Preeclampsia
NO: Non-treated
H/R: Hypoxia/reoxygenation
EXO: Exosomes
HGECs: Human glomerular endothelial cells
DOHaD: Developmental origins of health and disease
NTA: Nanoparticle tracking analysis
TEM: Transmission electron microscopy
