# Correction: Preeclampsia impedes foetal kidney development by delivering placenta-derived exosomes to glomerular endothelial cells

**DOI:** 10.1186/s12964-024-01697-5

**Published:** 2024-06-07

**Authors:** Mengqi Gu, Pengzheng Chen, Dongmei Zeng, Xiaotong Jiang, Qingfeng Lv, Yuchen Li, Fengyuan Zhang, Shuting Wan, Qian Zhou, Yuan Lu, Xietong Wang, Lei Li

**Affiliations:** 1 Department of Obstetrics and Gynecology, Shandong Provincial Hospital, Shandong University, Jinan, 250021 Shandong China; 2 Department of Obstetrics and Gynecology, Shandong Provincial Hospital Affiliated to Shandong First Medical University, Jinan, 250021 Shandong China; 3 The Laboratory of Medical Science and Technology Innovation Center (Institute of Translational Medicine), Shandong First Medical University (Shandong Academy of Medical Sciences) of China, Jinan, 250117 Shandong China; 4 Key Laboratory of Birth Regulation and Control Technology of National Health Commission of China, Shandong Provincial Maternal and Child Health Care Hospital, 328 Jingshi East Road, Jinan, 250025 Shandong China

**Correction: Cell Commun Signal 21, 336 (2023)**

**https://doi.org/10.1186/s12964-023-01286-y**

Following the publication of original article [1], the authors reported that the image for PE-exo in Fig. 2E and the panels G and H in Fig. 4 were incorrect, additionally the legend for Fig. 2K mistakenly included an extraneous letter 'K' in the description for NO-exo. The incorrect and corrected versions of these figures are presented below. The authors would like to apologise for any inconvenience caused.

Incorrect Fig. 2E and Fig. 2K

**Fig. 2:**
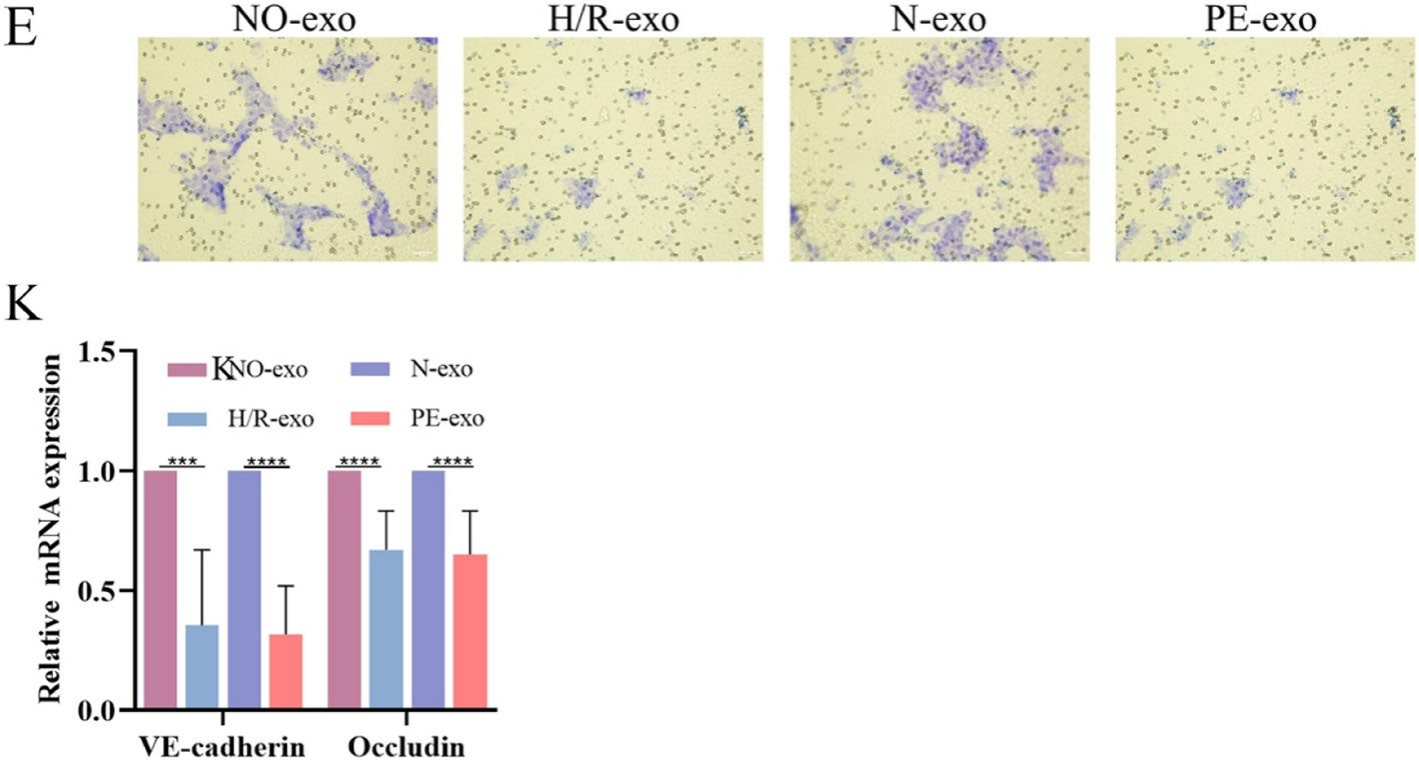
Function of trophoblast-derived exosomes on foetal mouse kidney.** E** Transwell experiments were used to examine HGECs migration after incubation with exosomes (100 μg/mL), Scale: 200 μm. **K** qRT-PCR of the expression of VE-cadherin and Occludin in exosomes

Correct Fig. 2E and Fig. 2K

**Fig. 2:**
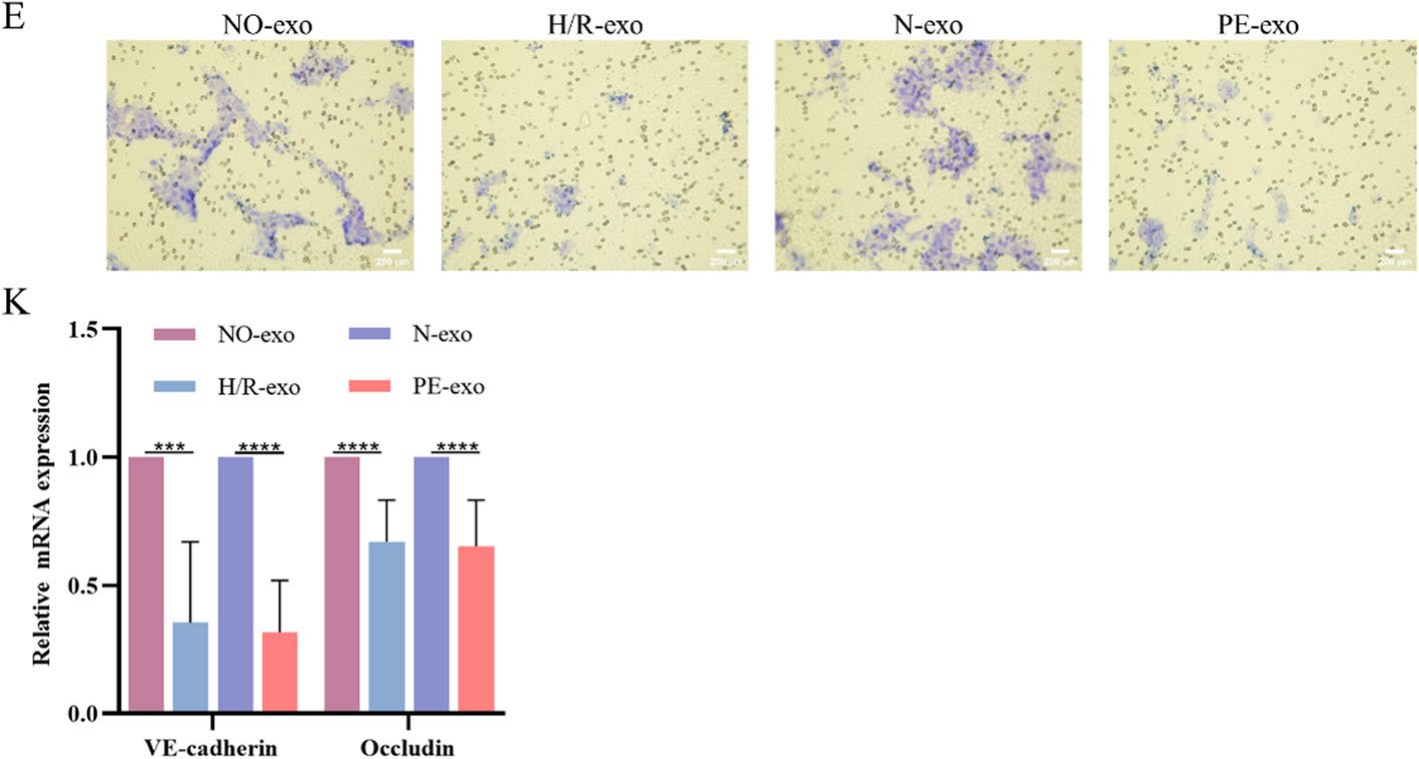
Function of trophoblast-derived exosomes on foetal mouse kidney.** E** Transwell experiments were used to examine HGECs migration after incubation with exosomes (100 μg/mL), Scale: 200 μm. **K** qRT-PCR of the expression of VE-cadherin and Occludin in exosomes

Incorrect Fig. 4G and Fig. 4H

**Fig. 4:**
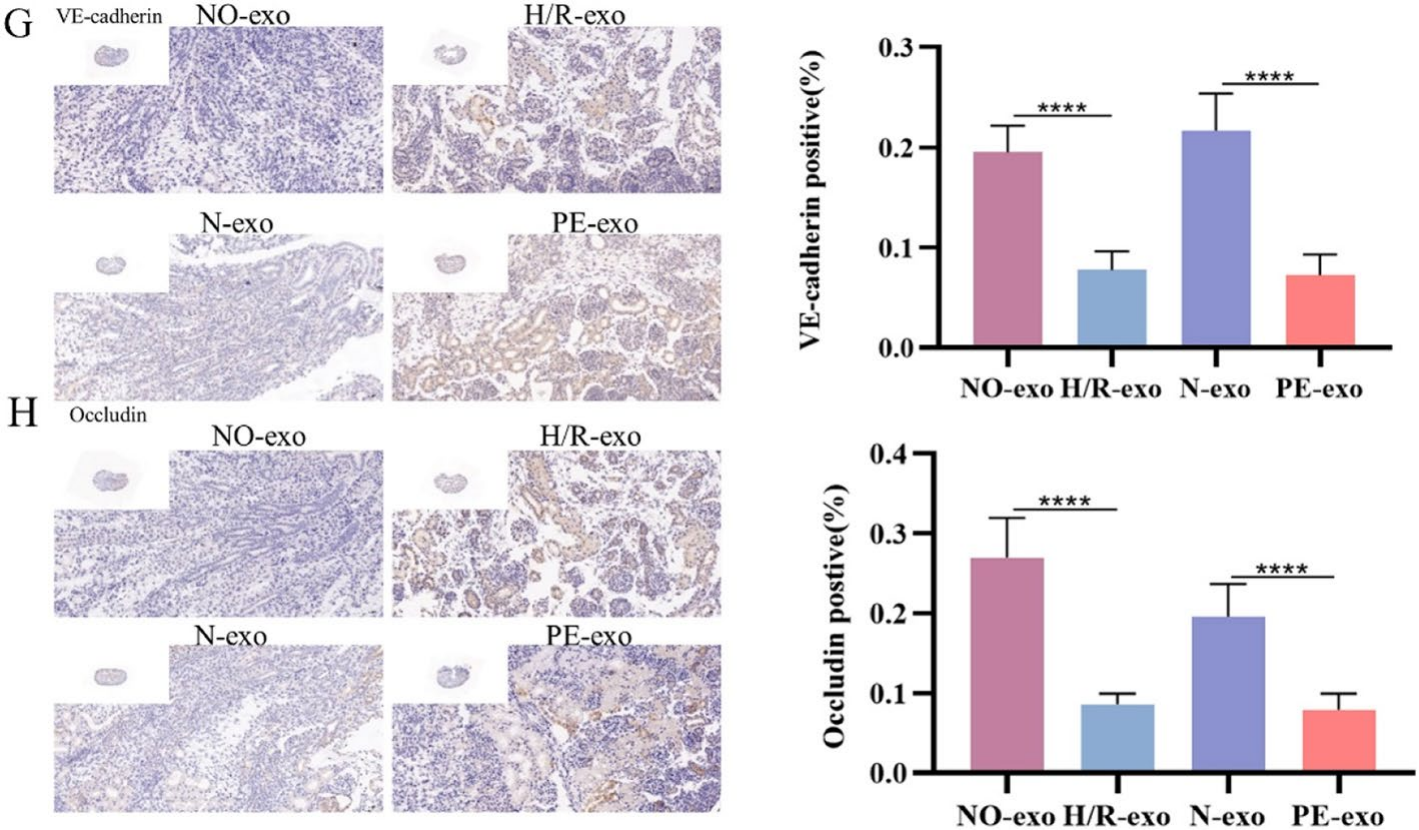
Function of trophoblast-derived exosomes on foetal mouse kidney. **G** IHC of the expression of VE-cadherin, Scale: 20 μm. **H** IHC of the expression of Occludin, Scale: 20 μm

Correct Fig. 4G and Fig. 4H

**Fig. 4:**
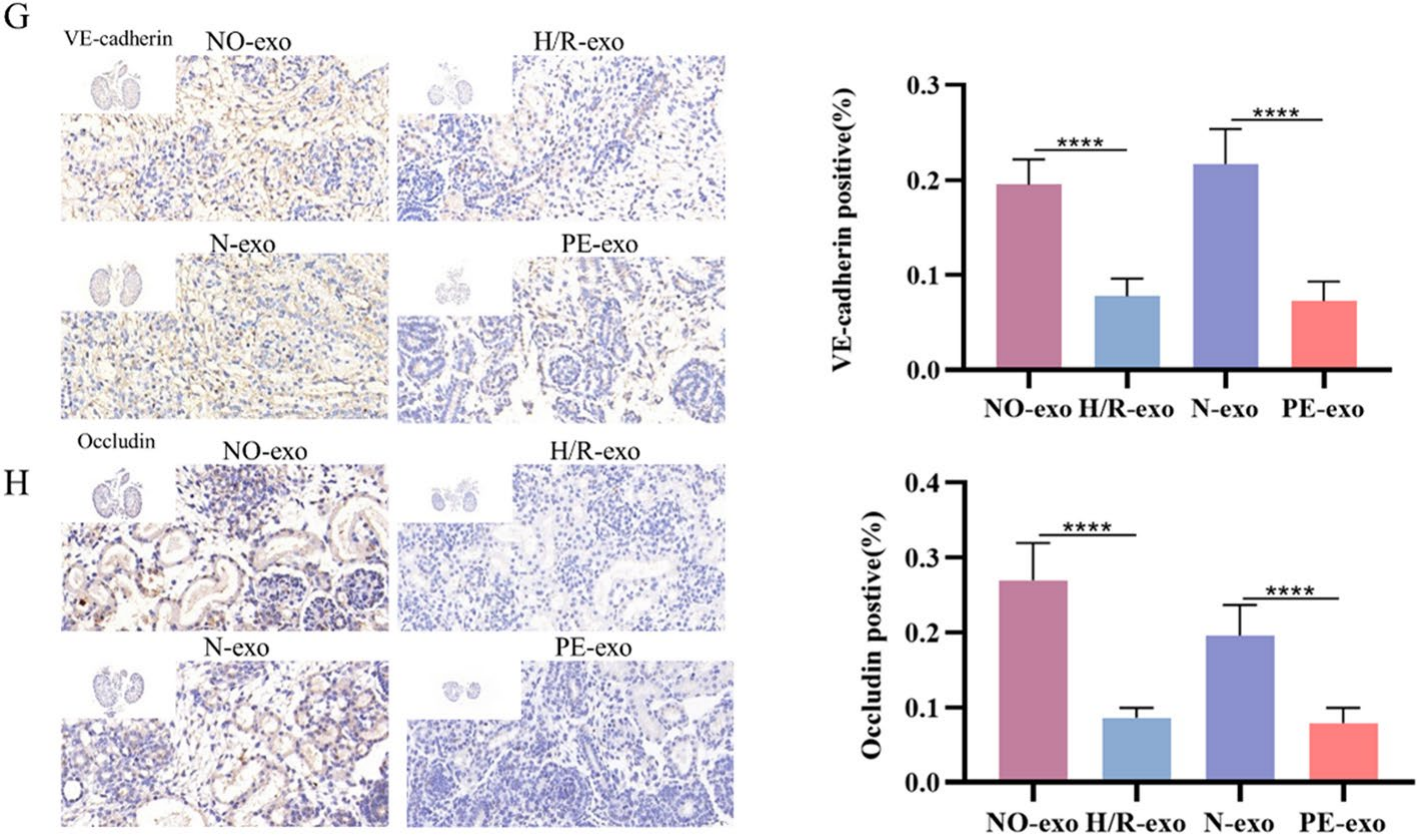
Function of trophoblast-derived exosomes on foetal mouse kidney. **G** IHC of the expression of VE-cadherin, Scale: 20 μm. **H** IHC of the expression of Occludin, Scale: 20 μm

We, the authors, confirm that the conclusions in the paper have not changed.
